# 3D micro-structured arrays of ZnΟ nanorods

**DOI:** 10.1038/s41598-017-02231-z

**Published:** 2017-05-18

**Authors:** Argyro N. Giakoumaki, George Kenanakis, Argyro Klini, Maria Androulidaki, Zacharias Viskadourakis, Maria Farsari, Alexandros Selimis

**Affiliations:** 1 IESL-FORTH, N. Plastira 100, 70013 Heraklion, Crete Greece; 2 Department of Chemistry, University of Crete, 70013 Heraklion, Crete Greece; 3 Crete Center for Quantum Complexity and nanotechnology, Physics Department, University of Crete, 70013 Heraklion, Crete Greece

## Abstract

The fabrication of nanostructures with controlled assembly and architecture is very important for the development of novel nanomaterial-based devices. We demonstrate that laser techniques coupled with low-temperature hydrothermal growth enable complex three-dimensional ZnO nanorod patterning on various types of substrates and geometries. This methodology is based on a procedure involving the 3D scaffold fabrication using Multi-Photon Lithography of a photosensitive material, followed by Zn seeded Aqueous Chemical Growth of ZnO nanorods. 3D, uniformly aligned ZnO nanorods are produced. The increase in active surface area, up to 4.4 times in the cases presented here, provides a dramatic increase in photocatalytic performance, while other applications are also proposed.

## Introduction

Zinc Oxide (ZnO) is a widely studied metal oxide semiconductor, due to its potential use in a variety of applications, such as gas sensors^1^, transparent electrodes in solar cells^2, 3^, phototocatalysts^4^, nanolasers^5^, photoelectrochemical cells for hydrogen generation from water splitting^6, 7^, photoluminescent devices^8, 9^, and organic light emitting diodes^10, 11^. Its useful properties, but also the plethora of geometries that can be grown (nanorods -NRs, nano-wires, nanobelts, nanosprings, hierarchical nanostructures etc.)^2, 12^ make it one of the most studied materials in nanoscience. Moreover, ZnO can be doped with metals that can tailor its optical, electrical or mechanical properties^6^.

For the fabrication of pure and doped ZnO nanostructures several chemical and physical synthesis methods have been adopted, including vapour-liquid-solid (VLS) method^5, 13^, chemical vapour deposition (CVD)^14^, thermal evaporation^15^, electrochemical deposition in porous membranes^16^, and aqueous chemical growth (ACG)^17^. Most of these growth techniques have been used to control the distribution of ZnO nanostructures on substrates such as glass, Si wafers, flexible organic films, wires, optical fibres, and inside polymeric 3D structures^18–22^. The patterning of ZnO nanostructures has been demonstrated mainly on flat surfaces.

**Figure 1:**
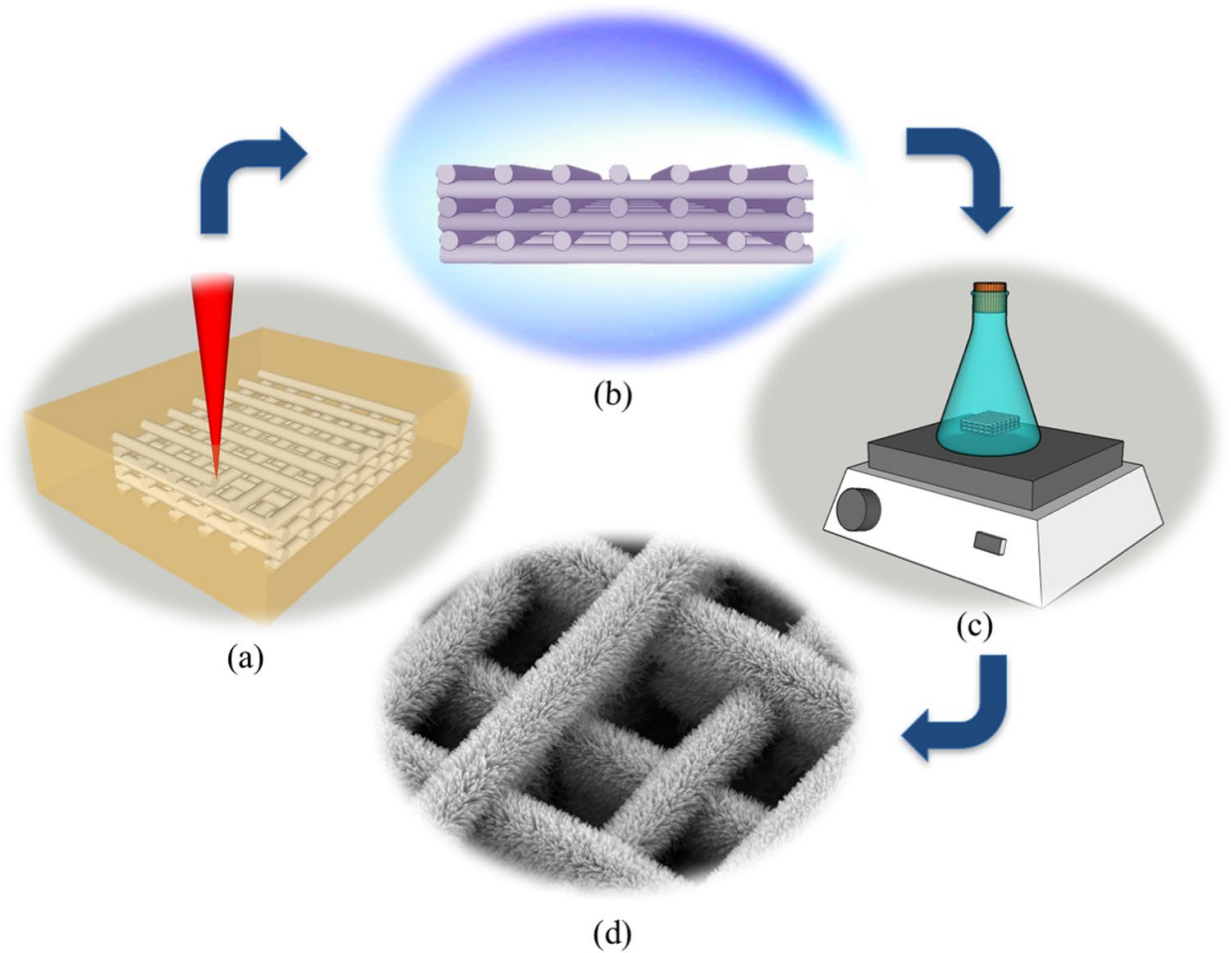
Experimental procedure for the fabrication of 3D micro-structured arrays of ZnΟ NRs. The 3D scaffolds fabricated by MPL (**a**) are coated with a Zn layer using PLD technique (**b**). The Zn coated scaffolds are subsequently chemically treated (**c**) for the ZnO NRs growth (**d**).

Here, we demonstrate an innovative method for the fabrication of, fully three-dimensional (3D) ZnO nanorods-coated structures, involving the seeded hydrothermal growth of ZnO NRs on a 3D scaffold of an organic-inorganic hybrid material (SZ2080), fabricated by Multi-Photon Lithography (MPL)^6^. The growth of ZnO NRs is based on a two-step procedure that requires the deposition of a metallic zinc (Zn) seed layer onto the polymeric scaffold, employing pulsed laser deposition (PLD), followed by an aqueous chemical growth of ZnO nanocrystalline rods out of an aqueous solution of zinc nitrate hexahydrate (Zn(NO_3_)_2_) in the presence of ammonia^23^. The proposed procedure is described graphically in Fig. 1.

The methodology presented is a straightforward and flexible scheme carried out at relatively low temperature (<100 °C), and is consistent with different type of substrates. Additionally, the laser-based techniques employed for the fabrication of 3D scaffold (MPL) and the seed Zn layer (PLD), allow the deposition of ZnO NRs in the form of micro-architected patterns on substrates with flat or complex geometry. Additional variability in the NR structure architecture can be introduced by varying the growth conditions (temperature, growth time etc.), while the incorporation of appropriate chemicals would enable the growth of doped or functionalized ZnO NRs^24^.

In what follows, we present ZnO nanostructured 3D architectures prepared with the proposed methodology. The properties of these structures, regarding morphology, structure, and fluorescence are examined. Additionally, their photocatalytic efficiency is investigated, and is found to increase dramatically compared to the efficiency of ZnO NRs of similar characteristics deposited on flat substrates. In the supplement, we demonstrate the essential role of the Zn seed layer for the uniform distribution of ZnO NRs over the surfaces of the assembly.

## Experimental Methods

### Sample Preparation

#### Scaffold material

The photopolymer used for the fabrication of the 3D scaffolds is the organic-inorganic hybrid coded as SZ2080, and is described in ref. 25. Its main components are [3-(Methacryloyloxy)propyl] trimethoxysilane (MAPTMS), methacrylic acid (MAA) and Zirconium n-propoxide (ZPO). As photoinitiator (PI) 4,4′-bis (diethylamino) benzophenone was used.

For the preparation of the composite, MAPTMS was hydrolysed using HCl solution (0.1 M) at a ratio of 1:0.1 and ZPO was stabilized by MAA (molar ratio 1:1). After 5 minutes, the Zirconium complex was slowly added to the hydrolysed MAPTMS at a 2:8 molar ratio. Finally, the PI, at a 1% mass ratio to the monomers was added to the mixture. After stirring for 15 minutes further, the composite was filtered using a 0.22 µm syringe filter.

The samples were prepared by drop-casting onto 100 micron-thick silanized glass substrates, and the resultant films were dried at 100 °C for 10 minutes before the photopolymerization. After the completion of the component laser writing process, the samples were developed for 20 minutes in a 30% v/v solution of 4-methyl-2-pentanone in isopropanol and were further rinsed with isopropanol.

#### Multiphoton Lithography (MPL)

The 3D scaffolds, serving us substrates for the growth of the ZnO NRs were prepared using the Multiphoton Lithography (MPL) method^26, 27^. This is a laser-based additive manufacturing technique, which allows the direct fabrication of fully 3D microstructures with high resolution. When the beam of ultrafast laser pulses is tightly focused inside the volume of a transparent, photopolymerisable resin, the high intensities within the focused beam voxel causes the absorption of more than one photons, resulting in local photopolymerision of the material. Moving the laser beam inside the material, 3D structures can be directly “written”; all that is needed afterwards is to remove the unexposed, unpolymerised resin, by immersing the sample into an appropriate solvent.

**Figure 2:**
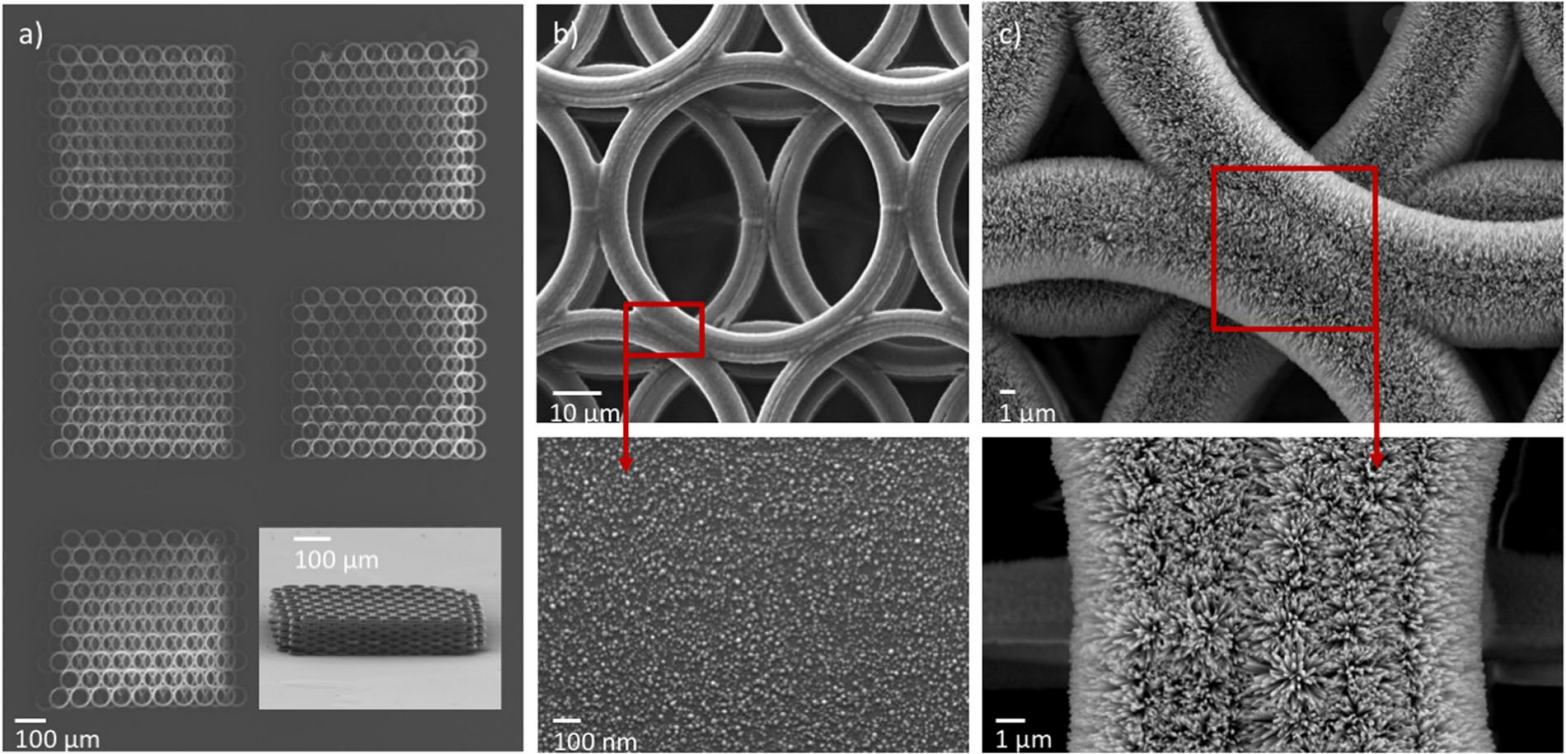
SEM images of a ZnO NRs decorated 3D array of circle stacks fabricated with MPL and Zn seeded ACG. (**a**) Section of pristine scaffolds of a the 3D array (**b**) circle stacks deposited with Zn (**c**) detail of the ZnO NRs chemically grown on the Zn-coated circles of the stacks. The scaffold has been fabricated with MPL technique using the galvanometric scanner system described in the experimental section (laser power = 35 mW, objective: 10x, scanning speed = 0.1 mm/s). ACG growth time: 2 h.

Two different experimental set-ups were employed in this work. The first system, thoroughly described in ref. 28, offers the fabrication of complex 3D structures at high speed (structures in Fig. 2). It comprises of a galvanometric scanner-based system (Scanlabs Hurryscan II, computer-controlled by SCAPS SAMLight software), where the focused laser beam is scanned through the polymeric sample to “write” a predetermined design, while the sample motion is restricted to the z-direction. The light source is a Ti:Sapphire femtosecond laser (Femtolasers Fusion, λ = 800 nm, 75 MHz, τ < 20 fs) and is focused into the photopolymerisable composite using focusing microscope objective lenses with high numerical aperture (100x, N.A. = 1.4, Zeiss, Plan Apochromat and 10x, N.A. = 0.45, Zeiss, Plan Apochromat). Before entering the scanner, the laser beam was expanded five times (5x) using a telescope lens to illuminate the full back aperture of the microscope objective and to achieve optimal focusing. Z-axis scanning and larger-scale x-y movements were possible by using a high-precision three-axis linear translation stage (Physik Instrumente). Beam on/off and power were further controlled by a mechanical shutter (Uniblitz) and a motorized attenuator (Altechna), respectively. The power used for the fabrication of the structures was 30–100 mW, measured before the objective, while the average transmission was 20%. The scanning speed was 100–20000 µm/s. The direct write process was monitored by a CCD camera mounted behind a dichroic mirror.

**Figure 3:**
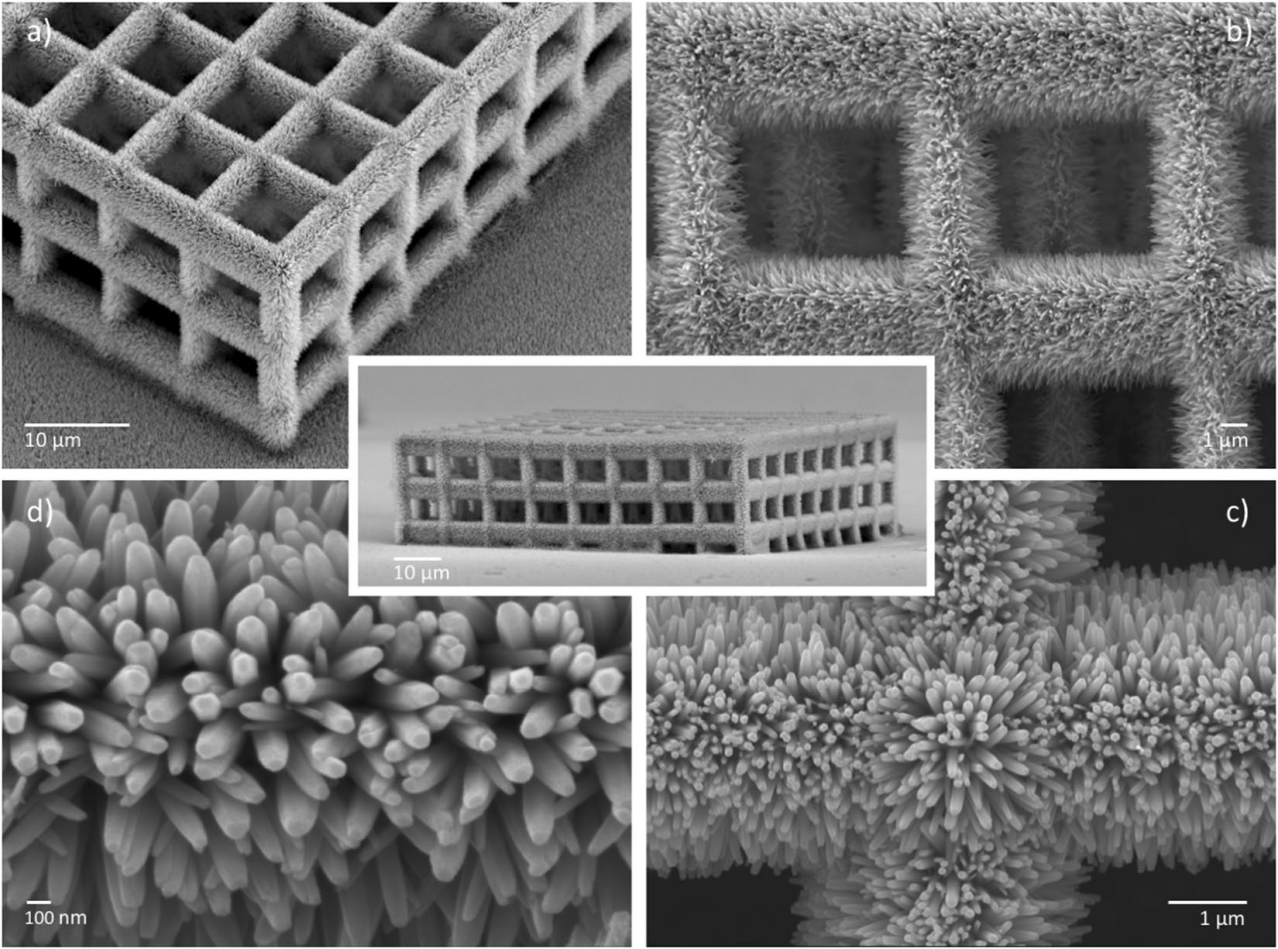
SEM images of a ZnO NRs coated 3D structure of blocks (center), fabricated with MPL and Zn seeded ACG. The scaffold has been fabricated with MPL using the xyz motion system described in experimental section (laser power = 10 mW, objective: 100x, scanning speed = 20 μm/s). ACG growth time: 2 h.

The second MPL system employed^29^, provides superior accuracy compared to the first one, but lower speed. It uses the same ultra-fast laser source and microscope objectives. For the fabrication of a polymeric scaffold, the laser beam remains immobile, while the sample moves using an xyz piezoelectric stages system, which allow fine and step movement (Physik Instrumente). This system was used to fabricate the structures of Fig. 3. The average power employed for the structure fabrication was 5–15 mW, measured before the objective, while the average transmission was 20%. The scanning speed was set to 10–20 µm/s.

#### Pulsed Laser Deposition (PLD)

The 3D scaffolds fabricated by MPL, were subsequently covered with a Zn thin film, acting as a seed layer for the chemical growth of ZnO nanostructures. The Zn thin film deposition was carried out using the PLD technique^19^, in high vacuum conditions (10^−7^ mbar) using standard PLD apparatus^30^. A UV laser beam (KrF excimer laser, λ = 248 nm, τ = 30 ns) was delivering a series of 2000 pulses at a repetition rate of 10 Hz. The beam was incident onto a bulk Zinc rotating target (Zinc foil 99.95%, GoodFellow) at an angle of 45° with respect to the target normal and was focused by a spherical lens (f = +30 cm) to yield a laser fluence of 3 J/cm^2^. The ablated material was collected on the 3D scaffolds, placed parallel to the target at a distance of 56 cm from the target surface. Deposition on flat substrates under identical conditions has resulted in Zn layers approximately 40 nm thick.

#### Aqueous Chemical Growth of ZnO NRs

Following Zn deposition, the Zn-seeded 3D scaffolds were chemically treated in a solution of 100 mL of 0.02 M aqueous solution of zinc nitrate hexahydrate Zn(NO_3_)_2_ · 6H_2_O (Sigma-Aldrich, 99.0%) and 3.1 mL ammonium hydroxide (28% wt NH_3_ in H_2_O, Fluka). More specifically, the Zinc-coated 3D structures were immersed vertically in the ammoniac zinc hydrate solution, to avoid deposition of ZnO nanorods due to gravity, under constant stirring at room temperature. The solution was gradually heated up to the temperature of 95 °C which kept stable for 2–5 hours. The as grown samples were rinsed with deionized water.

In order to compare the characteristics and the performance of the 3D micro-structured arrays, ZnO NRs were also grown on flat glass substrates coated with SZ2080 hybrid. The procedure followed for the growth of ZnO NRs was identical (Zn seeded ACG growth) to the one employed for growth on 3D scaffolds.

### Characterization

#### Scanning Electron Microscopy, X-Ray Diffraction and Photoluminescence

The Scanning Electron Microscope (SEM) images were recorded using the JEOL JSM-6390 LV model, at an accelerating voltage of 15 kV.

The ZnO NRs structural properties was deduced with X-ray diffraction (XRD), A Rigaku (RINT 2000) diffractometer with Cu Ka X-rays, at θ/2θ configuration, in the 2θ range of 30.00–70.00° was used.

Photoluminescence spectra were collected in air, at different temperature ranging from 14 K to room temperature, with a system comprising a He-Cd cw laser (325 nm, 2 mW) and a grating spectrometer (600 grooves/mm) equipped with a sensitive, liquid nitrogen-cooled CCD detector.

#### Photocatalytic activity studies

Many different methods can be used to determine the activity of photocatalytic surfaces. Popular techniques include those based on the photo-oxidation of organic films such as stearic acid^31, 32^, the decolourization of methylene blue (MB) in aqueous solutions^33, 34^, or contact angle changes^34^.

In this work, the photocatalytic activity of the ZnO NRs-coated 3D structures was quantified by measuring the decolourization of methylene blue (MB) in aqueous solution (initial concentration: 5.4 × 10^−7^ mol/L (20 ppm), pH: 5.5). This is a typical potent cationic dye, widely used as a model organic probe to test the photocatalytic performance of photocatalysts^33–35^. The samples were placed in a custom made quartz cell, and the whole setup was illuminated for up to 60 min using a UV lamp centred at 365 nm (Philips HPK 125 W), with a light intensity of ~10 mW/cm^2^. The MB concentration (decolourization) was monitored by UV-Vis spectroscopy in absorption mode, at the peak wavelength (λ_max_ = 665 nm), using a K-MAC spectrophotometer over the wavelength range of 220–800 nm.

The photocatalytic activity tests were carried out on five identical 3D nanostructured ZnO NRs samples, for five times on each sample, in order to examine the measurement reproducibility and the stability under UV illumination. For comparison, the photocatalytic performance of ZnO NRs, fabricated by the same growth procedure, on SZ2080-coated flat (glass) substrate of the same dimensions (5 mm × 5 mm) was also investigated. Blank experiments (photolysis) were also performed using glass (bare and SZ2080-coated) substrates under identical conditions with those applied for the ZnO samples.

## Results

Figure 2 depicts the SEM images of 3D array structures at different steps towards their decoration with ZnO nanorods, employing the proposed fabrication scheme. Figure 2a shows a part of a 3D array, prepared by MPL, consisting of parallel layers of stacked circles with approximately 50 μm diameter, which is next coated with metallic zinc, using PLD. The close-up image of the circles (Fig. 2b) confirms the uniform coverage with the Zn seed layer, consisting of nanoparticles having diameter of a few tens of nanometres. Following hydrothermal growth, vertically aligned ZnO NRs are grown to the regions where the zinc layer was initially deposited, as clearly shown in Fig. 2c.

Figure 3 shows SEM pictures of a different system, and particularly a ZnO NRs coated 3D periodic structure, consisting of parallel blocks of the same dimensions (10 × 10 × 10 μm^3^). The SEM images confirm the top-to-bottom uniformity and long-range continuity of the ZnO nanorod layer over the surfaces (Fig. 3a and c), even at the interior parts of the 3D assembly (Fig. 3b). The structure is covered by dense well-aligned ZnO nanorods, having diameter and length of 50 nm and 1 µm, respectively (Fig. 3d).

**Figure 4:**
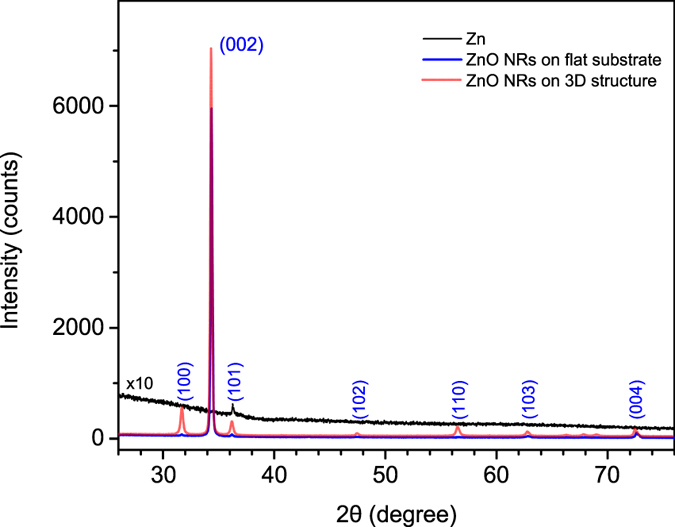
XRD pattern of Zn seed layer (black line), ZnO NRs grown on flat substrate (blue line) and on a 7 × 7 3D array of circle stacks (red line) depicted in Fig. 2.

The overall quality of the ZnO structures can be demonstrated via XRD analysis. Figure 4 presents the XRD pattern of ZnO nanostructures grown on flat substrates and 3D scaffolds. All the NRs arrays grown exhibit the θ/2θ XRD lines that can be indexed to the hexagonal würtzite structure of ZnO, in agreement with the JCPDS card file No. 36-1451. The strongest feature within the pattern is the (002) diffraction at about 34°, indicating preferential growth along [0001] crystallographic orientation. Τhe full width at half maximum (FWHM) of the (002) line is less than 0.2°, indicating a high-quality crystalline structure. Zn-coated glass substrates (dark curve in Fig. 4) revealed no diffractions peaks, implying that the XRD pattern of the as-grown samples is due to the ZnO nanostructures, while no impurities or other phases were detected.

**Figure 5:**
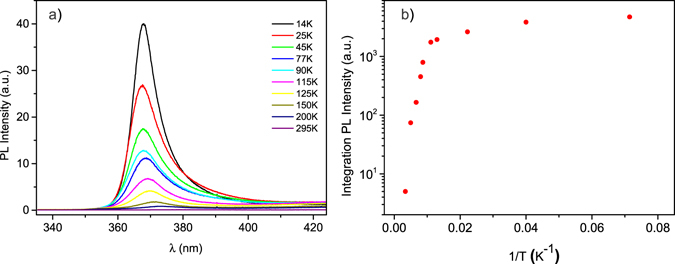
(**a**) PL emission spectra at different temperatures and (**b**) Arrhenius plotof plot of PL emission integrated intensity as a function of inverse temperature of a ZnO NRs-decorated 3D array of circle stacks, recorded in air with cw excitation at 325 nm (power: 2 mW).

An additional measure of the material quality was obtained from the photoluminescence (PL) emission of the ZnO NRs arrays. PL spectra were recorded upon excitation with a 325 nm continuous wave laser, at different temperatures, varying from 14 K to room temperature (Fig. 5). The strong UV photoluminescence emission band, centred at 380 nm at room temperature, is the characteristic near-band edge emission of the ZnO wide band-gap semiconductor^36^. With the temperature decrease, the PL emission is blue-shifted and takes the value of 367 nm at 14 K. From the Arrhenius plot integrated PL intensity as a function of inverse temperature T^−1^ (Fig. 5b), the activation energy of ZnO nanorods was estimated to be 60 meV, in agreement with previous reported observations^37^.

Taking into account the thickness of the samples (~1 μm), the conductivity of the ZnO nanostructured samples was calculated to be in the order of 10^−1^ S/m, using the method described in the supplement. This metallic behaviour is attributed to the hydrothermal growth of the ZnO NRs, during which hydrogen is available for n-type doping^38–43^. It could also be due to the presence of defects, oxygen vacancies as well as structural defects due to size, shape, and surface effects (polar surfaces causing charge transfer effects)^44^. It should be made clear that the conductivity will change as the sample thickness change, and this depends on the NR growth conditions.

The photocatalytic performance of the ZnO NR-decorated structures was studied by recording the degradation of methylene blue contaminant under UV irradiation. The decrease of MB concentration was recorded fοr 3D ZnO NRs coated assemblies of 5 × 5 mm^2^ overall area, possessing the complex geometry presented in Fig. 2, as well as on ZnO NRs deposited on flat substrates of the same area. The 3D ZnO NRs coated samples consisted of 7 × 7, 5 × 5, and 4 × 4 arrays of 49, 25 and 16 stacks of circles, respectively. Each stack comprised of 6 layers with 85 circles of 50 μm diameter each.

**Figure 6:**
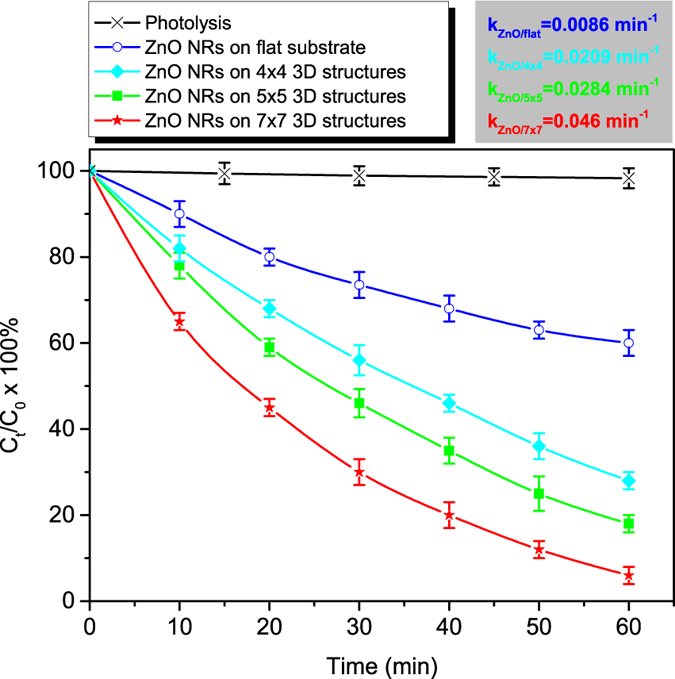
Methylene blue photodegradation over time under UV light irradiation of ZnO NRs grown on flat substrate and different 3D arrays of circle stacks presented in Fig. 3. The data correspond to measurements performed five times at three identical samples. The photolysis curve corresponds to data recorded on pristine flat glass substrates and 3D structures. The apparent rate constants (k) for each array are presented on the top of the diagram.

The time evolution of the MB concentration under the UV irradiation is shown in Fig. 6 for all tested samples. The photolysis curve (black curve in Fig. 6), recorded in the absence of the ZnO photocatalyst (pristine SZ2080-coated flat glass substrates and 3D structures), is also displayed for comparison. As clearly seen, the decrease of the dye concentration (from 100% to 96%) over the pristine structure within 120 min time interval is negligible, while a dramatic decrease occurs in the case of ZnO NRs-coated structures. This confirms that the MB photodegratation occurs onto the ZnO nanostructured material surface and is attributed to the reaction of MB with highly oxidative radicals (hydroxyl •OH and superoxide •${O}_{2}^{-}$) generated on the ZnO surfaces under UV irradiation^45^. As Fig. 6 illustrates, the ZnO NRs-coated 3D structures demonstrate higher reduction rate of MB concentration compared to the one onto the ZnO NRs grown on flat substrates. For example, the 7 × 7 arrays, which have a 4.4 times higher active area compared to the flat surfaces (see calculation in the supplement), induced the degradation of ~95% of the MB, after 60 min irradiation (even ~75% in only 30 min), while the ZnO nanorods on flat substrate provided only ~35% decolourization, after a period of 60 min.

In order to quantify the ZnO induced MB concentration decrease and the photocatalytic activity of the various structures the apparent rate constant (k) has been estimated, according to the Equation 1:

1 $$\mathrm{ln}({{\rm{C}}}_{{\rm{t}}}{/{\rm{C}}}_{{\rm{0}}})=-\,{\rm{kt}}$$

where C_0_ is the initial MB concentration, C_t_ is the concentration after a time interval t and k is specific rate constant for the first order kinetics reaction.

The good linear fit of the experimental data to Equation 1, confirms that the photodegradation of MB on ZnO nanostructures follows first-order kinetics. The 7 × 7, 5 × 5 and 4 × 4 arrays of ZnO NRs-coated stacks of circles provided apparent rate constants of 0.046 min^−1^, 0.0284 min^−1^, 0.0209 min^−1^ respectively, while the value corresponds to flat substrates is 0.0086 min^−1^. This is in agreement with previous results reported in literature^32, 33^ and can be justified on the basis of an optimum surface-to-volume ratio that yields a higher total surface available for interaction with MB, in the case of 3D ZnO samples^46^. Moreover the lower response of the flat samples can be attributed to the high jamming of the nanorods that in effect reduces the available absorption sites of MB on ZnO surface.

To further study the photocatalytic process, the photo-stability of the materials was tested by repeating the photo-degradation measurements five times on 3 identical samples. This study revealed that the material clearly preserves its photocatalytic properties and efficiency.

## Discussion

The key motivation of the work presented here is firstly to illustrate the principles of the fabrication scheme for the development of ZnO based 3D structures, with excellent morphological characteristics and physical properties. Secondly, to show that the many-fold enhanced active area of these new nanostructures allows for their integration in different technological fields and devices^6, 47–51^, such as photocatalysis, as it is demonstrated here, but also possibly in gas sensing, photo-detecting, or water splitting.

Moreover, the introduced methodology for the development of 3D nanostructured geometries requires the presence of a uniform thin film over the MPL written scaffold, which is deposited via a well-established, versatile deposition technique, PLD. This provides us with the confidence that the proposed fabrication scheme can be easily extended to a variety of materials and coatings such as metals, oxides, even polymers, as long as the hydrothermal growth temperature doesn’t exceed the temperature of 3D hybrid scaffold degradation (180 °C), opening the path for diverse new applications.

The disadvantages of this method are related to the slow speed and high cost of the MPL technique. However, MPL is the only available technique allowing the direct printing of free-form, complex 3D structures with the required resolution. Given the unique technology capabilities, there is a lot of concentrated research trying to improve its productivity by either developing faster photoinitiators, or by developing high-aspect ratio^52^ or holographic MPL^53, 54^, and this technology is progressing fast from the laboratory to the factory floor. If freeform structures are not needed, MPL is not necessary for the fabrication of 3D nanostructures. Another technique which could provide 3D periodic high-resolution polymeric structures is multi-photon interference lithography^55^. Using this technique, 3D periodic high-resolution structures could be made using only one (or a few) pulses of light; then the proposed methodology could be used to further functionalize the structures with zinc and ZnO.

## Conclusions

To summarize, here we have introduced an innovative approach for the reproducible fabrication of complex 3D ZnO NR structures, via combining MPL, a laser based direct writing technique, and a low temperature chemical growth (ACG), seeded by a Zn layer deposited by PLD. The structures exhibit excellent ZnO NRs covering and vertical alignment over the surfaces of the assemblies, as well as crystalline structure and electrical conductivity. We have also shown that the transition from 2D to 3D architectures results to a significant increase of the photocatalytic ability of the material, due to the increased active surface area and the multiaxial axial growth of ZnO NRs that enables the efficient absorption of the MP on the ZnO surface.

## Electronic supplementary material

Supplementary information
