# Supplementary material for: 3D micro-structured arrays of ZnΟ nanorods
Source: Sci Rep. 2017 May 18;7:2100. doi: 10.1038/s41598-017-02231-z (PMC5437041; doi:10.1038/s41598-017-02231-z)
Supplement: Supplementary file 1 — Supplementary information [file 41598_2017_2231_MOESM1_ESM.doc]

# Supplement

# 3D micro-structured arrays of ZnΟ nanorods

Argyro N. Giakoumaki,^1,2^ George Kenanakis,^1^ Argyro Klini,^1^

Maria Androulidaki,^1^ Zacharias Viskadourakis,^3^ Maria Farsari,^1^ and Alexandros Selimis^1^

*^1^IESL-FORTH, N. Plastira 100, 70013, Heraklion, Crete, Greece.*

*^2^Department of Chemistry, University of Crete, 70013, Heraklion, Crete, Greece.*

*^3^Crete Center for Quantum Complexity and nanotechnology, Physics Department, University of Crete, 70013, Heraklion, Crete, Greece.*

## ZnO rods grown without Zn seeding

In order to investigate the role of the Zn seed layer in the quality of ZnO NRs patterning, a number of pristine, without the Zn seed layer, 3D scaffolds were chemically treated in the growth solution to synthesize ZnO nanorods. Figure S1 shows the SEM images of a ZnO NRs covered 3D structure consisting of a number of columns heads (Figure S1a) fabricated with MPL. As evidenced, the grown ZnO features (Figure S1b) exhibit different characteristics compared to those grown on Zn coated 3D structures resembling a non-uniform, random growth throughout the deposited area, especially in the vertical side of the columns. Additionally, the formed ZnO NRs are of different shape and dimensions (Figure S1c). This is in agreement with a previous study underlying the significant role of the morphology of the Zn seed layer on the characteristics of the ZnO nanostructures grown over it.[^1^](#_ENREF_1)

| Figure S1: SEM images of ZnO NRs chemically grown on a periodic structure of columns, without the deposition of Zn seed layer (a), (b) Top and side view of the columns respectively, (c) ZnO NRs on the top of the column. The scaffold has been fabricated by MPL technique using the galvanometric scanner system described in the materials and methods section (laser power=35 mW, objective: 10x, scanning speed=0.1 mm/s). ACG growth time: 3 h |
| --- |

## Calculation of the increase in the active surface area for the 7x7 circle samples

To estimate approximately the increase in the active surface area, we calculated the surface Area (*A_r_*) of each ring using the torus surface area equation:


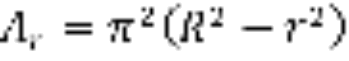


where *r= 0.021 mm* and *R=0.028 mm* are the inner and outer diameter of the torus, respectively. Using these numbers, we find that each ZnO NR-coated ring has an active surface area 0.0034 mm^2^ and multiplying it with the number of rings ((85 on each layer of the stack) x (6 layers) x (49 stacks)), we find that the total ring active surface area is approximately 85 mm^2^. The flat sample active surface area *A_f_* =5 mm x 5mm = 25 mm^2^. The total increase in the surface are is therefore


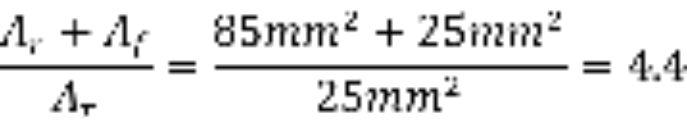


The 3D-structured sample has therefore 4.4 times more active surface area, i.e. more ZnO NRs for catalysis.

### Electrical Properties

For the electrical characterization of the ZnO NR structures, DC Current–Voltage experiments were performed at room temperature. Conductive Ag paste was used and metallic contacts were built on opposite sides of the sample, as shown in Figure S2, resembling a two-probe device. A Keithley 2400 Source-Meter was used to measure the current passed through the device, with respect to the applied voltage.

| 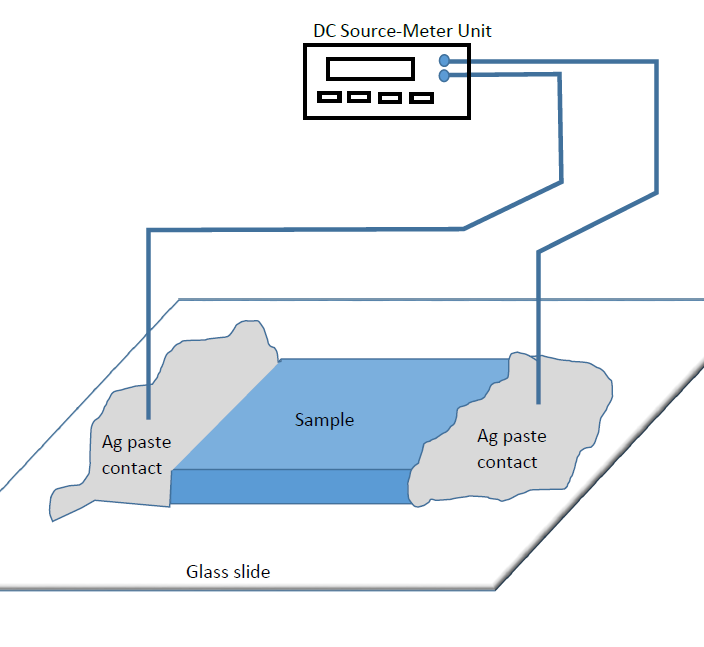 | |
| --- | --- |
|  |  |
| Figure S2: Schematic diagram of the geometry used for electrical measurements. | |

# References

1 Claeyssens, F., Klini, A., Mourka, A. & Fotakis, C. Laser patterning of Zn for ZnO nanostructure growth: Comparison between laser induced forward transfer in air and in vacuum*. Thin Solid Fil*m**s 5**15, 8529-8533 (2007).
